# Hh/Gli antagonist in acute myeloid leukemia with CBFA2T3-GLIS2 fusion gene

**DOI:** 10.1186/s13045-017-0396-0

**Published:** 2017-01-21

**Authors:** Riccardo Masetti, Salvatore Nicola Bertuccio, Annalisa Astolfi, Francesca Chiarini, Annalisa Lonetti, Valentina Indio, Matilde De Luca, Jessica Bandini, Salvatore Serravalle, Monica Franzoni, Martina Pigazzi, Alberto Maria Martelli, Giuseppe Basso, Franco Locatelli, Andrea Pession

**Affiliations:** 1 Department of Pediatrics, “Lalla Seràgnoli”, Hematology-Oncology Unit, Sant’Orsola-Malpighi Hospital, University of Bologna, Via Massarenti 11, 40137 Bologna, Italy; 2 “Giorgio Prodi” Cancer Research Center, University of Bologna, Bologna, Italy; 3 Institute of Molecular Genetics, National Research Council-IOR, Bologna, Italy; 4 Department of Biomedical and Neuromotor Sciences, University of Bologna, Bologna, Italy; 5 Department of Woman and Child Health, Hematology-Oncology, University of Padova, Padova, Italy; 6 Department of Pediatric Hematology-Oncology, IRCCS Ospedale Bambino Gesù, Rome, Italy; 7 University of Pavia, Pavia, Italy

**Keywords:** Acute myeloid leukemia, Acute megakaryoblastic leukemia, CBFA2T3-GLIS2, GANT61, Hedgehog pathway

## Abstract

**Background:**

*CBFA2T3*-*GLIS2* is a fusion gene found in 17% of non-Down syndrome acute megakaryoblastic leukemia (non-DS AMKL, FAB M7) and in 8% of pediatric cytogenetically normal acute myeloid leukemia (CN-AML, in association with several French-American-British (FAB) subtypes). Children with AML harboring this aberration have a poor outcome, regardless of the FAB subtype. This fusion gene drives a peculiar expression pattern and leads to overexpression of some of *Hedgehog*-related genes. GLI-similar protein 2 (GLIS2) is closely related to the GLI family, the final effectors of classic Hedgehog pathway. These observations lend compelling support to the application of GLI inhibitors in the treatment of AML with the aberration *CBFA2T3*-*GLIS2*. GANT61 is, nowadays, the most potent inhibitor of GLI family proteins.

**Methods:**

We exposed to GANT61 AML cell lines and primary cells positive and negative for *CBFA2T3*-*GLIS2* and analyzed the effect on cellular viability, induction of apoptosis, cell cycle, and expression profile.

**Results:**

As compared to AML cells without *GLIS2* fusion, GANT61 exposure resulted in higher sensitivity of both cell lines and primary AML cells carrying *CBFA2T3*-*GLIS2* to undergo apoptosis and G1 cell cycle arrest. Remarkably, gene expression studies demonstrated downregulation of *GLIS2*-specific signature genes in both treated cell lines and primary cells, in comparison with untreated cells. Moreover, chromatin immunoprecipitation analysis revealed direct regulation by GLIS2 chimeric protein of *DNMT1* and *DNMT3B*, two genes implicated in important epigenetic functions.

**Conclusions:**

Our findings indicate that the GLI inhibitor GANT61 may be used to specifically target the *CBFA2T3*-*GLIS2* fusion gene in pediatric AML.

**Electronic supplementary material:**

The online version of this article (doi:10.1186/s13045-017-0396-0) contains supplementary material, which is available to authorized users.

## Findings

Pediatric acute myeloid leukemia (AML) carrying *CBFA2T3*-*GLIS2* fusion gene deserves particular interest, being associated with a grim prognosis in all the reports published so far [1–3]. The incidence of this aberration is 17 and 8% in pediatric non-Down syndrome acute megakaryoblastic and in pediatric cytogenetically normal AML, respectively [1–3]. The expression profile of *CBFA2T3*-*GLIS2* is associated with upregulation of both Hedgehog (HH) and bone morphogenic protein (BMP) signaling [1, 4].

The protein GLIS2 shares a highly homologous zinc finger domain with members of the GLI proteins, the final effectors of classic Hedgehog pathway. GANT61 is a GLI inhibitor showing a potent effect on the inhibition of transcription activity of GLI proteins, blocking their binding to DNA [5–8]. Considering the high homology of the DNA-binding domain between GLIS2 and GLI family proteins, we hypothesized that GANT61 might be used to specifically target the *CBFA2T3*-*GLIS2* fusion gene in pediatric AML.

In the present study, we investigated the in vitro effects of GANT61 on AML cell lines and primary cells from AML patients harboring the *CBFA2T3*-*GLIS2* fusion gene.

The materials and methods are detailed in Additional file 1. Molecular analysis of *CBFA2T3*-*GLIS2* fusion gene is reported in Additional file 2: Figure S1. Genetic features of control AML cell lines without GLIS2 fusion are reported in Additional file 3: Table S1.

**Fig. 1:**
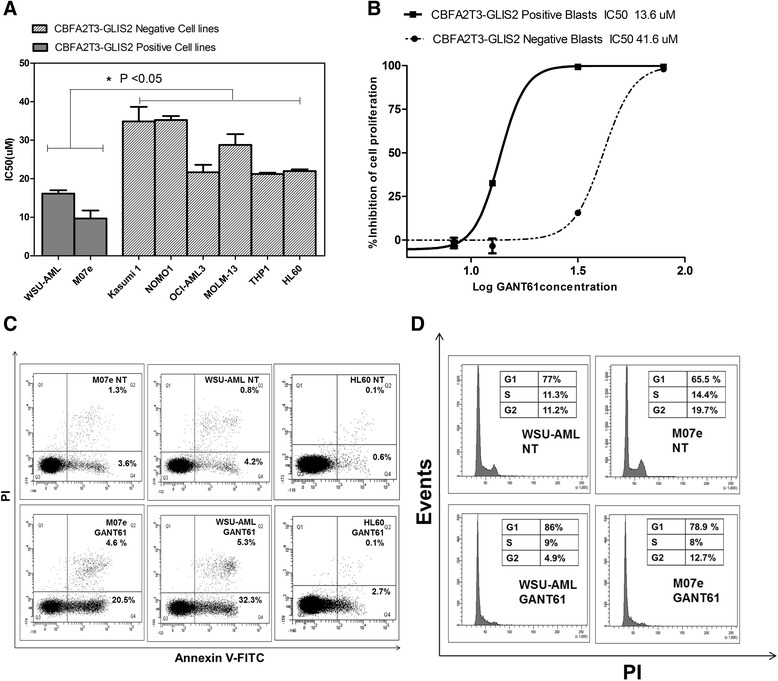
**a** IC50 of *CBFA2T3*-*GLIS2* and negative cell lines 72 h after GANT61 exposure. **b** Dose-response curves after 72 h of GANT61 treatment of primary cells derived from patients with acute myeloid leukemia (AML) either positive or negative for *CBFA2T3*-*GLIS2* fusion gene. *p < 0.05 **c** Flow cytometric analysis of Annexin V FITC/PI-stained AML cell lines treated for 24 h with 20 μM of GANT61. The percentages of early apoptotic cells (Annexin V FITC^+^/PI^−^, *lower right quadrant*) and late apoptotic/necrotic cells (Annexin V FITC^+^/PI^+^, *upper right quadrant*) are indicated. *NT* sample treated with vehicle alone (DMSO). **d** Cell cycle analysis. Flow cytometric analysis of PI-stained AML cell lines carrying the *CBFA2T3*-*GLIS2* fusion gene after 48 h of treatment with GANT61. *NT* sample treated with vehicle alone (DMSO)

Our results showed that AML cell lines with *CBFA2T3*-*GLIS2* fusion gene have a higher sensitivity to GANT61 than other AML cell lines without this genetic aberration (Fig. 1a). Similar results were obtained on primary leukemia cells isolated from AML patients, being the IC_50_ of the *GLIS2*-positive leukemia and negative primary cells 13.6 and 41.6 μM, respectively (Fig. 1b).

Treatment with GANT61 induced an increase of about 30% of apoptotic cells (Fig. 1c) and block of cell cycle in G0/G1 phase only in M07e and WSU-AML lines positive to *CBFA2T3*-*GLIS2* (Fig. 1d and Additional file 4: Figure S2).

**Fig. 2:**
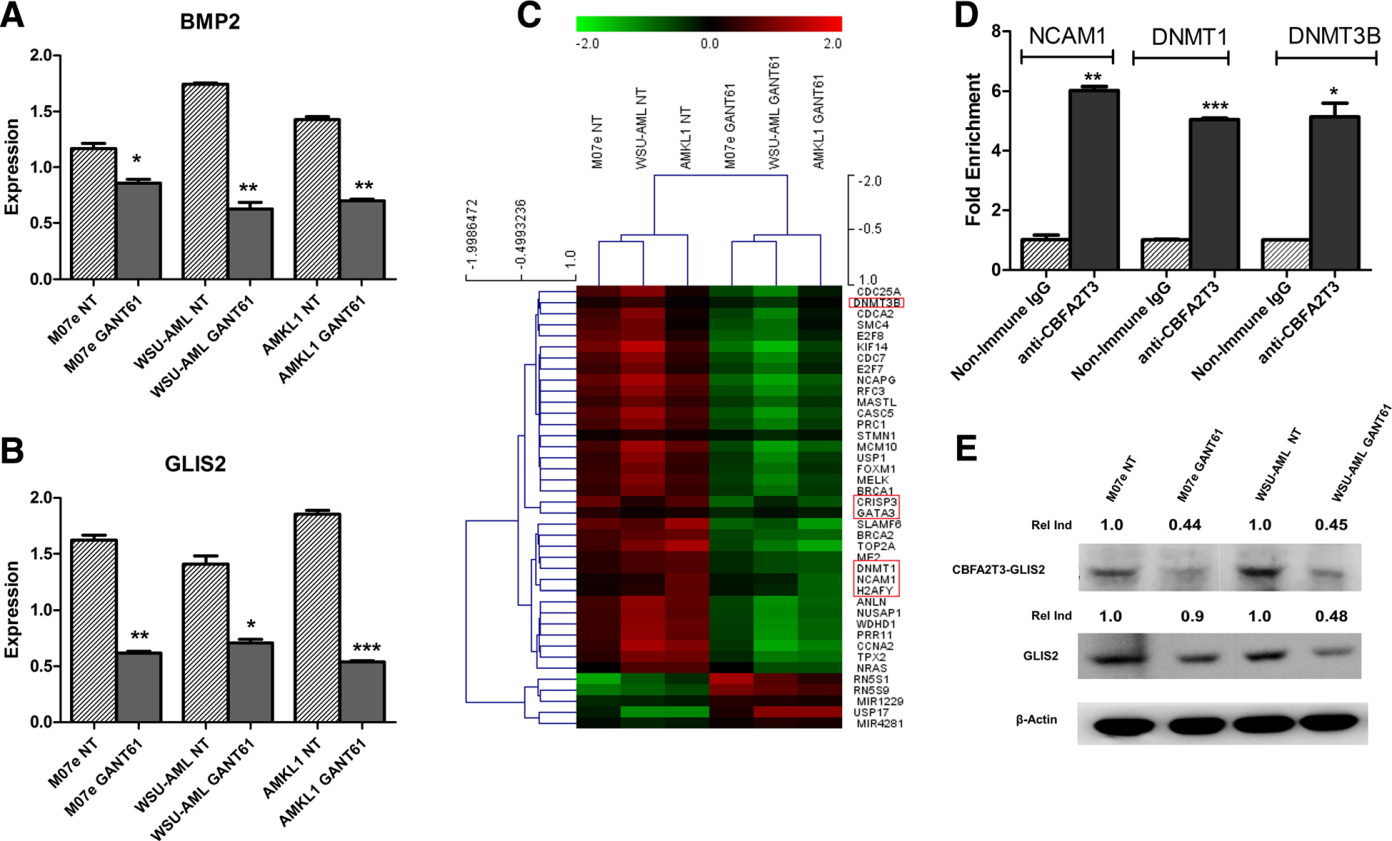
Quantitative PCR of selected mediators of *GLIS2* pathway **a** *BMP2* and **b** *GLIS2* after 48 h treatment with GANT61. *p < 0.05; **p < 0.01; ***p < 0.001. **c** Hierarchical clustering of genes differentially expressed between untreated and treated AML leukemia blast cells and cell lines, with a *p* value <0.05. **d** ChIP analysis, performed on WSU-AML cell line showed around five-fold enrichment of chimeric protein on *DNMT1*, *DNMT3B*, and *NCAM1* promoters. **e** Western blot analysis showing the decrease of GLIS2 protein and CBFA2T3-GLIS chimeric protein in samples treated with GANT61. Thirty micrograms of protein were blotted to each lane. Antibody to β-actin served as a loading control. The Relative Induction (Rel Ind) is the amount of protein present in treated samples relative to untreated cells after normalizing to β-actin density

We further analyzed the expression profile of cell lines and primary cells following GANT61 treatment. Through qPCR, we demonstrated that GANT61 treatment led to a significant reduction of the expression of *BMP2* and *GLIS2* (Fig. 2a, b). In order to fully characterize the effect of GANT61 treatment on whole transcriptome profile of *CBFA2T3*-*GLIS2*-positive cells, gene expression was assessed by microarray analysis. After GANT61 treatment, the expression of target genes of *CBFA2T3*-*GLIS2*, such as *CRISP3*, *GATA3*, *H2AFY*, or *NCAM1*(CD56) was significantly downregulated (*p* < 0.05) (Fig. 2c). Moreover additional genes were downregulated by the treatment with GANT61 (*p* < 0.05), as for example, genes involved in cell cycle control, such as *CDC25A*, *CDC7*, *CDCA2*, and *CCNA2*. The expression of these genes is required for progression through cell cycle, and their expression is aberrant in AML, as well as in other malignancies [9, 10]. In our model, the expression of these genes is downregulated following GANT61 treatment, this partly explaining the cell cycle arrest observed in AML cell lines with GLIS2 fusion after GANT61 treatment (Fig. 1d). Genes involved in cell proliferation (*KIF14*, *MELK*, *MCM10*, *NUF2*) and epigenetic regulators namely *DNMT1* and *DNMT3B* were also present. Considering the particular interest of these DNA methyltransferase genes, we performed chromatin immunoprecipitation (ChIP) analysis using a CBFA2T3-specific antibody on WSU-AML and M07e cell lines. Our findings showed that CBFA2T3-GLIS2 fusion protein directly binds to the proximal promoter of *DNMT1* and *DNMT3B*, positively regulating their expression (Fig. 2d). Overexpression of DNMT genes could lead to DNA hypermethylation and could be involved in the leukemogenesis process.

Since in the classic Hedgehog signaling pathway, several target genes involved in feedback mechanisms (*HHIP*, *PTCH1*, *GLI1*) have been described; we hypothesized that CBFA2T3-GLIS2 chimeric protein could regulate wild-type GLIS2 protein with the same feedback mechanism. Western blotting analysis confirmed the presence of chimeric protein and showed also the presence of wild-type GLIS2. These results may point to the role of the fusion protein in regulating wild-type GLIS2. Following GANT61 treatment, the expression of both proteins was decreased with respect to untreated samples, suggesting that GANT61 treatment targeted both CBFA2T3-GLIS2 fusion protein and also wild-type GLIS2 expression (Fig. 2e). Moreover, other AML cell lines not carrying the *CBFA2T3*-*GLIS2* fusion gene did not show expression of GLIS2 (data not shown).

On the other hand, western blotting analysis showed that expression of GLI1 and GLI2 did not decrease following GANT61 treatment (Additional file 5: Figure S3).

Another study demonstrated a high sensitivity of this subgroup of AML with GLIS2 fusion to Aurora A kinase (AURKA) inhibitors [4]. We therefore investigated the effect of GANT61 and AURKA inhibitor MK-0457 in M07e and WSU-AML cell lines carrying the *CBFA2T3*-*GLIS2* fusion gene. Cell lines were incubated for 48 h with either single drugs or a combination of the two drugs at a constant ratio of 1:10 (GANT61:MK-0457). The combined treatment showed a higher cytotoxic effect when compared to each single drug, and the two inhibitors displayed a synergistic effect on cell growth, as indicated by the CI value (Additional file 6: Figure S4).

This work provides preliminary data from preclinical in vitro and ex vivo studies targeting pediatric AML with *CBFA2T3*-*GLIS2* fusion gene. Although further investigation will be required to confirm these results, our experience with GANT61 represents a preliminary background for further evaluating in vivo the inhibition of *GLIS2*-mediated transcription in AML harboring the *CBFA2T3*-*GLIS2* fusion gene.

## Additional files

Additional file 1: Materials and methods. (DOCX 22 kb)
Additional file 2: Figure S1. A) Molecular analysis of *CBFA2T3*-*GLIS2* fusion gene. 1.M07e, 2.WSU-AML, 3.KASUMI1, 4.NOMO1, 5.OCI-AML3, 6.MOLM13, 7.THP1, 8.HL60, 9.Negative Control. B) Sequencing of *CBFA2T3*-*GLIS2* fusion gene in M07e and in C) WSU-AML. (TIF 6759 kb)
Additional file 3: Table S1. Genetic features of control AML cell lines. * Data provided by DSMZ and by literature. (XLSX 11 kb)
Additional file 4: Figure S2. Cell cycle analysis. Flow cytometric analysis of PI-stained AML cell lines negative to *CBFA2T3*-*GLIS2* fusion gene after 48 h of treatment with GANT61. NT: sample treated with vehicle alone (DMSO). (TIF 183 kb)
Additional file 5: Figure S3. Western blotting analysis of GLI1/2 protein in cell lines either positive or negative for *GLIS2* fusion gene treated with GANT61. NT, untreated cells. One representative of three independent experiments is shown. (TIF 205 kb)
Additional file 6: Figure S4. Cytotoxic effect of either GANT61, or MK-0457 or of GANT61 in association with MK-0457 on A) M07e and in B) WSU-AML cell lines. (TIF 369 kb)

## Abbreviations

AML: Acute myeloid leukemia
AURKA: Aurora A kinase
BMP: Bone morphogenic protein
ChIP: Chromatin immunoprecipitation
FAB: French-American-British
GLIS2: GLI-similar protein 2
HH: Hedgehog
PI: Propidium iodide
WST1: 4-[3-(4 lodophenyl)-2-(4-nitrophenyl)-2H-5-tetrazolio]-1,3-benzene disulfonate)
