# Supplementary material for: Hh/Gli antagonist in acute myeloid leukemia with CBFA2T3-GLIS2 fusion gene
Source: J Hematol Oncol. 2017 Jan 21;10:26. doi: 10.1186/s13045-017-0396-0 (PMC5251306; doi:10.1186/s13045-017-0396-0)
Supplement: Additional file 1: — Materials and methods. (DOCX 22 kb) [file 13045_2017_396_MOESM1_ESM.docx]

**MATERIALS AND METHODS**

**Cell Lines and reagents**

Human AML cell lines (M07e, HL60, NOMO1, Kasumi-1, MOLM-13, THP-1, OCI-AML3) were obtained from DSMZ; WSU-AML cell line was kindly provided by St. Jude Children's Research Hospital, Memphis, TN, USA. WSU-AML, HL60, NOMO1, MOLM13, THP1 and KASUMI-1 were grown in RPMI 1640 (Lonza, Veviers, Belgium), M07e cells were grown in Iscove Medium Dulbecco’s modified (IMDM) (Sigma, St Louis, USA) and OCI-AML3 in Alpha-MEM (Lonza, Veviers, Belgium). Cell lines and primary cells with *CBFA2T3-GLIS2* fusion gene were FAB M7. All culture media were supplemented with 10% Fetal Bovine Serum (EuroClone, Milan, Italy), 2mM L-glutammine (NY, USA) and 100 U/ml penicillin/streptomycin (NY, USA). Primary cells were obtained from two pediatric patients with FAB M7 AML (AMKL1) positive to *CBFA2T3*-*GLIS2* and FAB M4 negative for the same translocation, respectively. Primary cells were grown in Chang Marrow (Irvine Scientific, Santa Ana, USA).

**Cell viability assay**

To test the effect of GANT61 (Merck Millipore, Darmstadt, Germany), cell lines were cultured for 72 hours in the presence of increasing (from 0.1 µM to 100 µM) drug concentrations. To test the combination of Aurora A Kinase inhibitor MK-0457 and GANT61 we treated M07e and WSU-AML at a constant ratio of 1:10 (GANT61:MK-0457) for 48 hours. Cell viability was assessed using the WST1 (4-[3-(4-lodophenyl)-2-(4-nitrophenyl)-2H-5-tetrazolio]-1,3-benzene disulfonate), cell proliferation kit (Roche Diagnostic, Basel, Switzerland), according to manufacturer’s instructions. The curve and IC_50_ were calculated by non-linear dose-response regression with Graphpad Prism software (San Diego, CA, USA). Statistical analyses were performed using Student’s *t* test at a significance level of p <0.05 (GraphPad Prism Software). For drug-combination experiments, a combination index (CI) number was calculated using the Compusyn software. CI values **<**1 defined different grades of synergism, values 1 corresponded to an additive effect, whereas values > 1.1 were indicative of antagonism.

**Cell cycle analysis and apoptosis assay**

Cell cycle analysis was performed staining cells with propidium iodide (PI) solution (PI 50 μg/ml; 0.1% sodium citrate; 0,1% Triton X-100) according to standard procedures [15] and after 48 hours of treatment with 20µM of GANT61.

To assess the extent of induced apoptosis, flow cytometric analysis of Annexin V–FITC/propidium iodide (PI)-stained samples was performed after 24 hours of treatment with 20µM of GANT61 and in basal condition, following manufacturer's instruction (Annexin-V-FLUOS Staining Kit, Roche Diagnostics, Monza, Italy). Samples were analyzed by a FACSCanto flow cytometer (Beckton Dickinson, Franklin Lakes, NJ). Apoptotic cells were identified as Annexin V-positive, PI-negative cells.

**Western Blotting**

Western blotting analysis was performed using standard procedures after 48 hours of treatment with 20µM of GANT61. Cells were lysed using M-PER Mammalian Protein Extraction Reagent, supplemented with the Protease and Phosphatase Inhibitor Cocktail (Thermo Fisher Scientific Inc., Rockford, IL). GLIS2 antibody was purchased from Santa Cruz Biotechnology (Heidelberg, Germany), GLI1 antibody was purchased from Cell Signaling Technology and GLI2 was purchased from Biorad. Secondary antibodies were purchased from Cell Signaling Technology. Densitometry scanning of the bands was performed using a Chemidoc 810 Imager with the appropriate software (UVP, Upland, CA).

**Quantitative PCR, gene expression and molecular analysis of *CBFA2T3-GLIS2* fusion gene.**

Cell lines (M07e, WSU-AML) and primary cells (AMKL1) carrying the *CBFA2T3*-*GLIS2* fusion gene were grown in presence of GANT61 20 µM or vehicle (DMSO) for 48 hours. Total RNA was extracted by RNeasy spin column method (Qiagen, Hilden, Germany). 250 ug of RNA was reverse transcribed to single-stranded cDNA using the Transcriptor first strand cDNA synthesis kit (Roche Diagnostics, Monza, Italy) with oligo-dT primers (2.5 μM). qRT-PCR was performed with FastStart Sybr Green (Roche Diagnostics, Monza, Italy) on the LightCycler 480 apparatus (Roche Diagnostics, Monza, Italy) using specific primers for *BMP2, GLIS2.* DDCt method was used to quantify gene expression levels relative to two housekeeping genes, *YWHAZ* and *ATP5B*. Quantitative RT-PCR primer sequences were as follows:

*GLIS2* Forward primer 5' GAGGTTTCAACGCCAGGTACA 3' Reverse primer 5' GCAGACGTAGGGCTTCTCAC 3', *BMP2* Forward primer 5' GACTGCGGTCTCCTAAAGGTCG 3’ Reverse primer 5' CTGTTTCAGGCCGAACATGC 3’, *ATP5B* Forward primer 5' GTCTTCACAGGTCATATGGGGA 3’ Reverse 5’ GTCCCACCATATAGAAGG 3’, *YWHAZ* 5’ AAAAACAGCAGATGGCTCGAGAA 3’ Reverse 5’ GTGAAGCATTGGGGATCAAGAAC 3’.

Gene expression profile was assessed using GeneChip Human Transcriptome Array 2.0 (Affymetrix, Santa Clara, CA, USA). Microarray target sample processing, target hybridization, washing, staining and scanning steps were completed according to manufacturer's instructions (Affymetrix).

Data normalization and summarization was performed using the RMA (Robust Multi-Array Average) method [16]. Before proceeding with differential expression computation, the dimensions of the dataset were reduced by filtering out genes whose IQR is smaller than the 10^th^ percentile of global IQR and whose expression level is below 5 in more than two samples. To score the differences of expression between the two conditions, we used the moderated t-statistics for paired samples (implemented in *limma* package) with a significance level α =0.05. Differentially expressed genes were then classified as either upregulated (logFC>1) or downregulated (logFC< 1).

For molecular analysis of *CBFA2T3*-*GLIS2* fusion gene, cDNA of AML cell lines was amplified with specific primers: CBFA2T3 Forward 5’CGAAGGGCCTCAGCTAGACGT3’ and GLIS2 Reverse 5’ATCCAAATAGCGCAGTGGCT3’. We included in the analysis the amplification of Beta Actin (Primer Forward: 5’ TCACCCACACTGTGCCCATCTACGA 3’, Primer Reverse: 5’ CAGCGGAACCGCTCATTGCCAATGG 3’) to test the efficiency of RNA Retro-transcription. PCR product was purified and labeled with BigDye Terminator 1.1 (Applied Biosystems, California, USA) and sequenced on a ABI3730 instrument (Applied Biosystems, California, USA).

**ChIP analysis**

ChIP analysis was performed using the chromoFlash High sensitivity ChIP kit (Epigentek, NY, USA). In brief, 1 × 10^6^ of WSU-AML and M07e cell lines were fixed with 1% formaldehyde, lysed, and sonicated (Bioruptor Pico sonication device, Diagenode, Seraing, Belgium). Sheared chromatin was immune-precipitated for 3h using the following antibodies: anti-CBFA2T3 (Abcam, Cambridge, UK), and normal rabbit non-immune IgG (Epigentek, NY, USA). One tenth of the sheared chromatin (Input) was used as a reference for the quantitative PCR analysis using the primers proximal NCAM1 promoter (forward, 5′-GCATCTGCCTCCCTGTCTCT-3′; and reverse, 5′-CTCGCAACTCGGAGATCCTT 3′), *DNMT1* (forward 5’ TACTCAAGGGCTCTCACAAA 3’ and reverse 5’CGAGGCATTCATTCATTCAT3’) and *DNMT3B* promoter (forward 5’ GGGCTACAAGGGGAGT3’ and reverse 5’GCTCGGAGCGTCCAC3’) with FastStart Sybr Green (Roche Diagnostics, Monza, Italy) on the LightCycler 480 apparatus (Roche Diagnostics, Monza, Italy).
