# Supplementary figures and images for: Hh/Gli antagonist in acute myeloid leukemia with CBFA2T3-GLIS2 fusion gene
Source: J Hematol Oncol. 2017 Jan 21;10:26. doi: 10.1186/s13045-017-0396-0 (PMC5251306; doi:10.1186/s13045-017-0396-0)

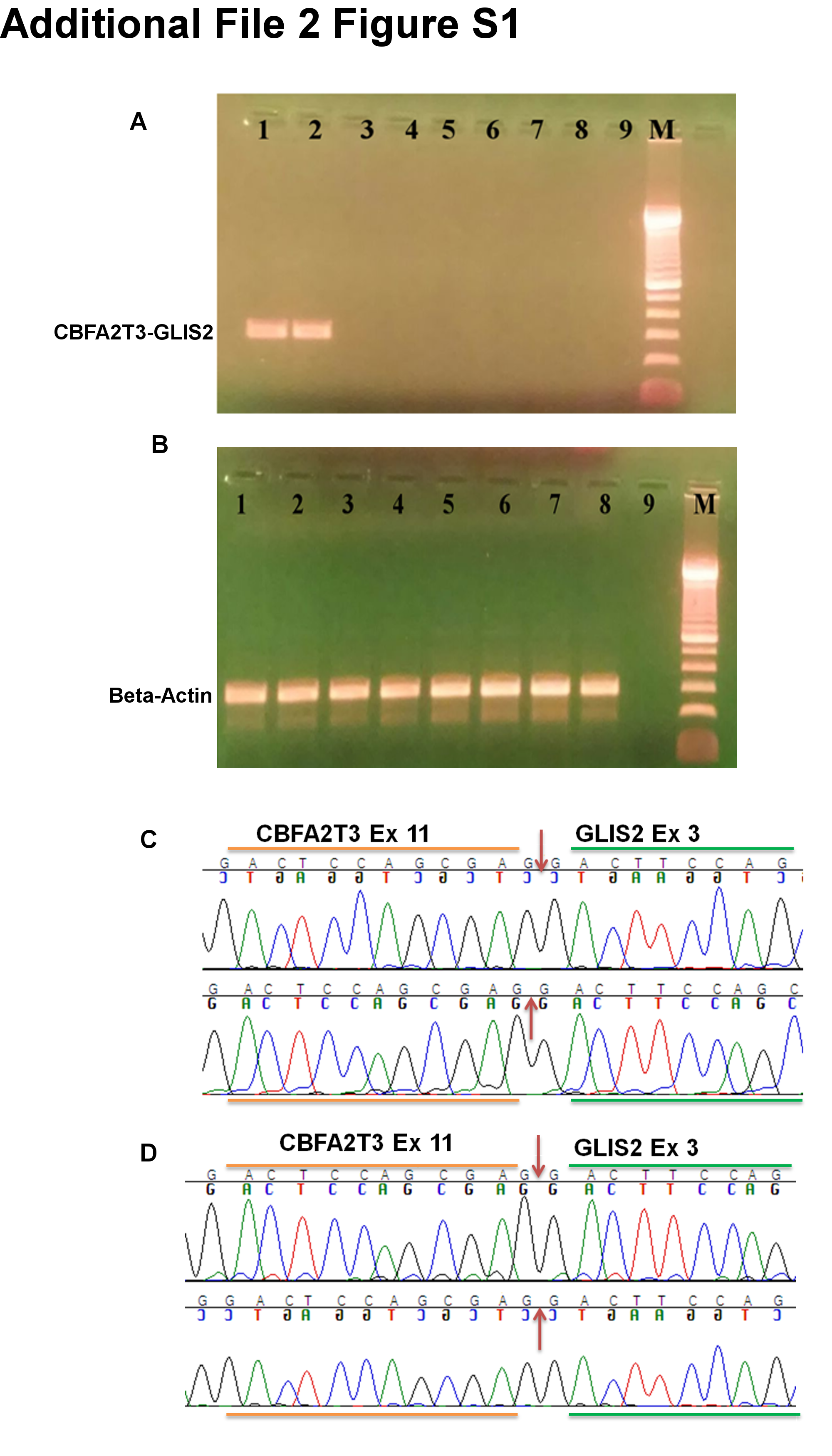

Supplement: Additional file 2: Figure S1. — A) Molecular analysis of CBFA2T3-GLIS2 fusion gene. 1.M07e, 2.WSU-AML, 3.KASUMI1, 4.NOMO1, 5.OCI-AML3, 6.MOLM13, 7.THP1, 8.HL60, 9.Negative Control. B) Sequencing of CBFA2T3-GLIS2 fusion gene in M07e and in C) WSU-AML. (TIF 6759 kb) [file 13045_2017_396_MOESM2_ESM.tif]

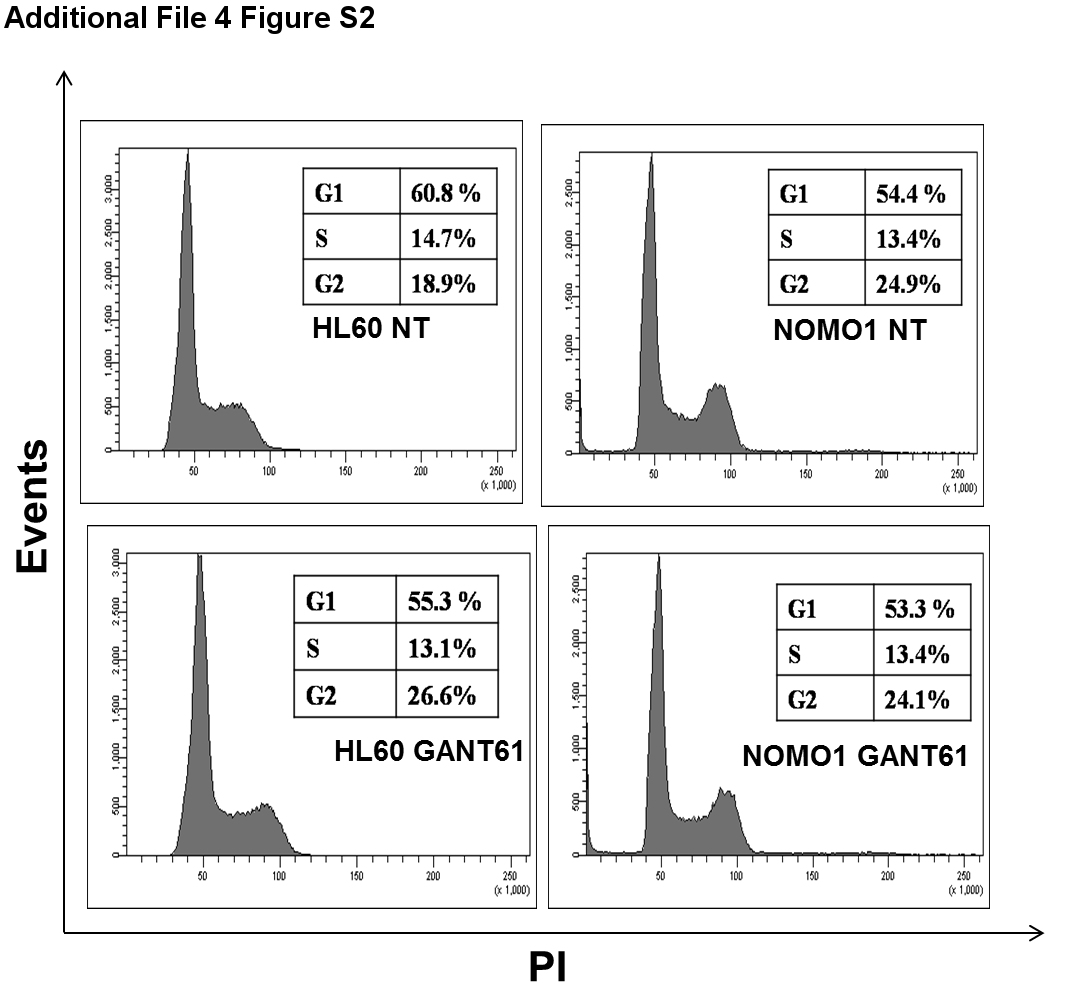

Supplement: Additional file 4: Figure S2. — Cell cycle analysis. Flow cytometric analysis of PI-stained AML cell lines negative to CBFA2T3-GLIS2 fusion gene after 48 h of treatment with GANT61. NT: sample treated with vehicle alone (DMSO). (TIF 183 kb) [file 13045_2017_396_MOESM4_ESM.tif]

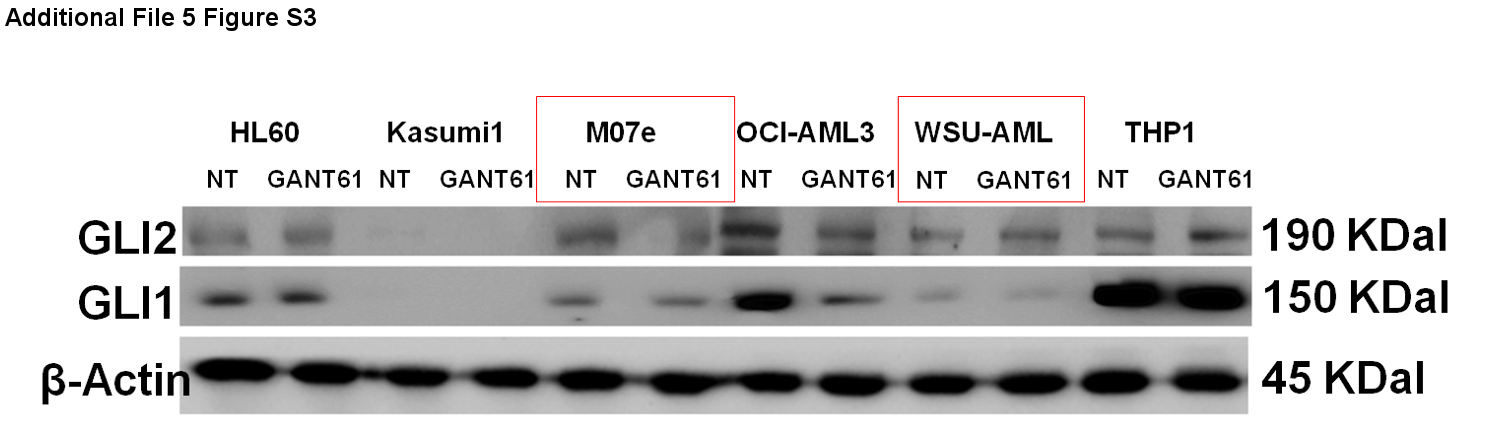

Supplement: Additional file 5: Figure S3. — Western blotting analysis of GLI1/2 protein in cell lines either positive or negative for GLIS2 fusion gene treated with GANT61. NT, untreated cells. One representative of three independent experiments is shown. (TIF 205 kb) [file 13045_2017_396_MOESM5_ESM.tif]

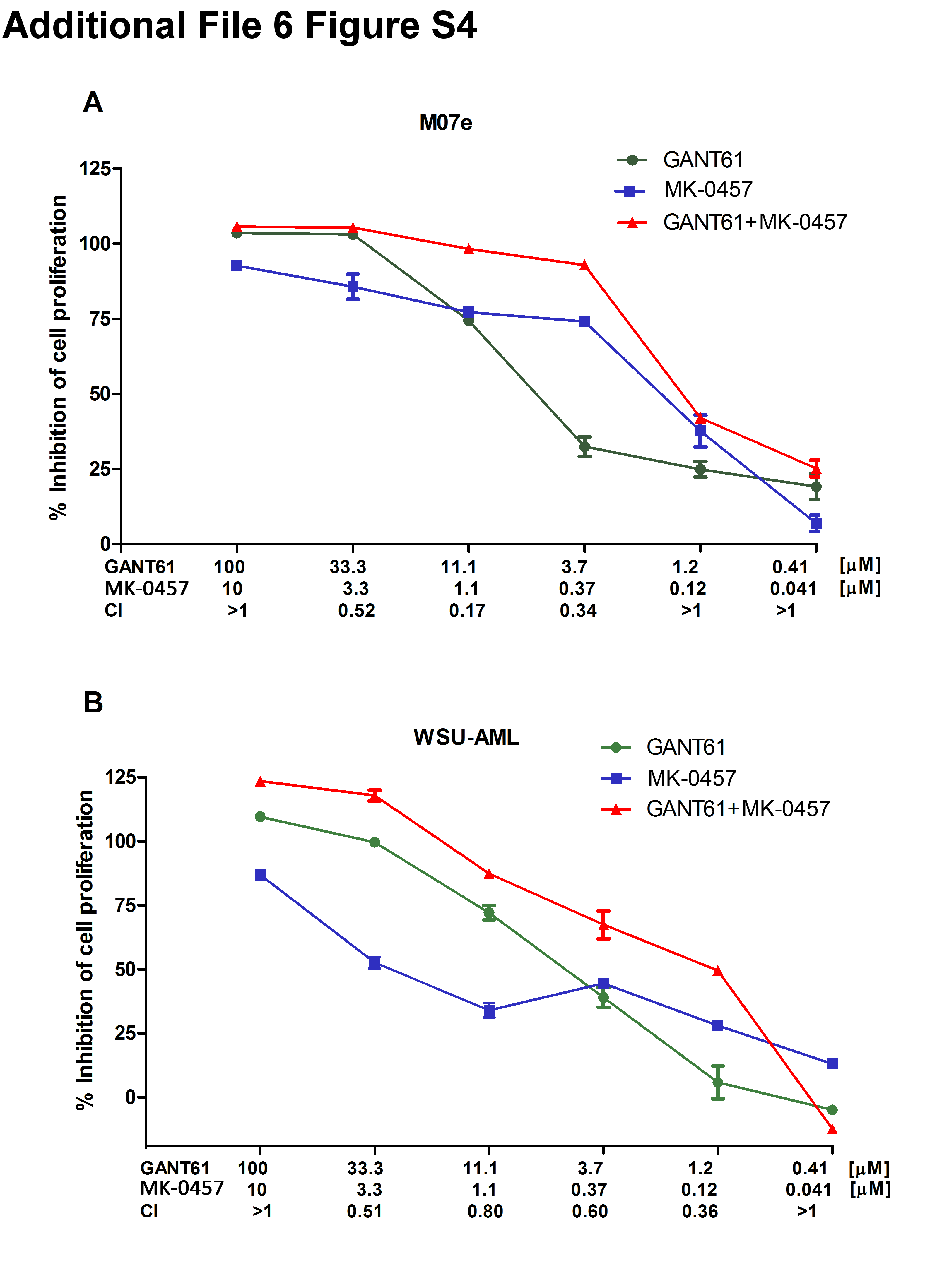

Supplement: Additional file 6: Figure S4. — Cytotoxic effect of either GANT61, or MK-0457 or of GANT61 in association with MK-0457 on A) M07e and in B) WSU-AML cell lines. (TIF 369 kb) [file 13045_2017_396_MOESM6_ESM.tif]
